# Association of reduced retinal arteriolar tortuosity with depression in older participants from the Northern Ireland Cohort for the Longitudinal Study of Ageing

**DOI:** 10.1186/s12877-021-02009-z

**Published:** 2021-01-15

**Authors:** R. A. O’Neill, A. P. Maxwell, F. Kee, I. Young, R. E. Hogg, S. Cruise, B. McGuinness, G. J. McKay

**Affiliations:** Centre for Public Health, Queens University Belfast, Belfast, Northern Ireland

**Keywords:** Retinal microvascular parameters, Depression score, Centre for epidemiological studies- depression, CES-D, NICOLA

## Abstract

**Introduction:**

The retina shares similar anatomical and physiological features with the brain and subtle variations in retinal microvascular parameters (RMPs) may reflect similar vascular variation in the brain. The aim of this study was to assess associations between RMPs and measures of depression in the Northern Ireland Cohort for the Longitudinal Study of Ageing.

**Methods:**

RMPs (arteriolar and venular caliber, fractal dimension and tortuosity) were measured from optic disc centred fundus images using semi-automated software. Depression was characterised by the Centre for Epidemiologic Studies Depression Scale (CES-D) in the absence of mild cognitive impairment or use of anti-depressive medications. Associations between depression and RMPs were assessed by regression analyses with adjustment for potential confounders.

**Results:**

Data were available for 1376 participants of which 113 (8.2%) and 1263 (91.8%) were classified with and without depression. Participants had a mean age of 62.0 ± 8.4 yrs., 52% were female, and 8% were smokers. Individuals with depression had a higher CES-D score than those without (22.0 ± 6.2 versus 4.4 ± 3.9). Lower values of arteriolar tortuosity were significantly associated with depression, before and after adjustment for potential confounders (odds ratio = 0.79; 95% confidence intervals: 0.65, 0.96; *P* = 0.02).

**Conclusion:**

Decreased retinal arteriolar tortuosity, a measure of the complexity of the retinal microvasculature was associated with depression in older adults independent of potential confounding factors. Retinal measures may offer opportunistic assessment of microvascular health associated with outcomes of depression.

**Supplementary Information:**

The online version contains supplementary material available at 10.1186/s12877-021-02009-z.

## Introduction

Depression is a serious, common mental health condition that incorporates feelings of sadness or hopelessness necessitating clinical intervention. Depression prevalence increases with age and is associated with higher levels of morbidity, suicide, self-neglect and reduced physical, cognitive and social functioning [1–4]. As lifespans increase, identification of those at greater risk of age-related conditions, including depression, becomes increasingly important to ensure appropriate provision of treatment [5, 6].

The most rapidly expanding age demographic in Northern Ireland (NI) are those aged 50 years (yrs) and above. The NI population provides a unique opportunity for evaluation of depression in a setting historically shaped by conflict and an undertone of sectarian violence [7, 8]. ‘The Troubles’ was a period of conflict that spanned from 1969 to 1994 and resulted in increased psychological morbidity and mental health problems among individuals exposed to higher levels of conflict [9–11]. During this time, a five-fold increase in anti-depressant prescriptions was reported in the NI population (1989–2000), especially among those aged > 55 yrs. who were three times more likely to be prescribed anti-depressant medication than the 15–24 yrs. demographic [9], highlighting the increasing burden of depression among the local older population.

The retinal microvasculature shares similar anatomical and physiological features with other end organs including the brain, heart and kidneys, and subtle variations in retinal microvascular parameters (RMPs) may reflect similar variation within the cerebral, renal and coronary circulation [12–14]. Recent advances in retinal fundus imaging provide a unique and non-invasive assessment of the microvasculature currently not possible elsewhere in the body [15–18], enabling opportunistic evaluation of vascular disease and the wider systemic circulation [13–18].

Variation in RMPs have been previously reported in association with cognitive impairment, depression and cerebrovascular disease [19–22] although the nature of associations between RMPs and depression have been inconsistent [23–27]. A ‘vascular depression’ hypothesis has previously implicated cerebrovascular disease in the predisposition, precipitation, and perpetuation of geriatric depressive syndromes [28]. As such, the aim of this study was to assess associations between RMPs and measures of depression in the Northern Ireland Cohort for the Longitudinal Study of Ageing (NICOLA).

## Methods

### Participants

NICOLA is a longitudinal cohort study consisting of 8468 adults aged 50 yrs. and over, located in Northern Ireland (those resident in care homes or other residential institutions at baseline were excluded from the study) [7]. The study included a computer-aided personal interview (CAPI), a self-completion questionnaire (https://static-content.springer.com/esm/art%3A10.1186%2Fs12882-020-02031-0/MediaObjects/12882 2020 2031 MOESM2 ESM.pdf) and approximately 45% completed a health assessment. The CAPI was extensive in scope and included assessment of demographic, social and health-related factors, and was conducted at individual home appointment between December 2013 and March 2016. Measures of cardiovascular, physical, cognitive and visual function were determined and biological samples collected, including visual health with retinal fundus photography. Written informed consent was obtained prior to participation following ethical approval from the School of Medicine, Dentistry and Biomedical Sciences Ethics Committee, Queen’s University Belfast (SREC 12/23) and in accordance with the Helsinki Declaration.

### Measurement and classification of depression and co-variates

Participants undertook the Centre for Epidemiologic Studies Depression Scale questionnaire (CES-D) which consisted of 20 questions scored from 0 to 3, to assess depressive symptoms with summative scores ranging between 0 and 60 [29]. Cognitive function was assessed using the 30 point Montreal Cognitive Assessment (MoCA) in addition to questions on subjective cognitive decline (SCD) and difficulties associated with basic activities of daily living (ADL), such as dressing, walking, bathing or showering, eating, getting in or out of bed, and using the toilet. Use of anti-depressive medications was defined as use of psycholeptic drugs (Anatomical Therapeutic Chemical [ATC] classification N05) and/or psychoanaleptic drugs (ATC classification N06). Mean arterial blood pressure (MABP) was an average of two individual systolic (SBP) and diastolic blood pressure (DBP) measurements (2/3 DBP + 1/3 SBP). Diabetes was characterised by HbA1c ≥48 mmol/mol, use of diabetic medications or self-reported diabetes. Cardiovascular disease (CVD) was characterised by self-reported history of angina, heart attack, congestive heart failure or stroke. Participant height was measured to the nearest centimetre using a seca 240 wall mounted measuring rod and weight was measured in kilograms using seca electronic floor scales. High and low-density lipoprotein (HDL and LDL) was measured from individual participant blood samples. Educational attainment was dichotomised as primary schooling and below or secondary level and above (including university education). Smoking status was classified as current smokers versus non-smokers. Alcohol consumption was categorised as: non-drinker, light drinker (0–7 units per week), moderate drinker (7–14 units per week) and heavy drinker (> 14 units per week). Exclusion criteria included those individuals who failed to complete the CES-D questionnaire, were classified with mild cognitive impairment (MCI; defined as MoCA test score ≤ 26 and SCD, in the absence of depression or problems with ADL activities), were taking anti-depressive medication or had retinal images or insufficient quality (Supplementary Figure 1). Depression was defined as a CES-D score ≥ 16 in the absence of MCI or anti-depressive medication use.

### Measurement of retinal microvascular parameters

**Fig. 1:**
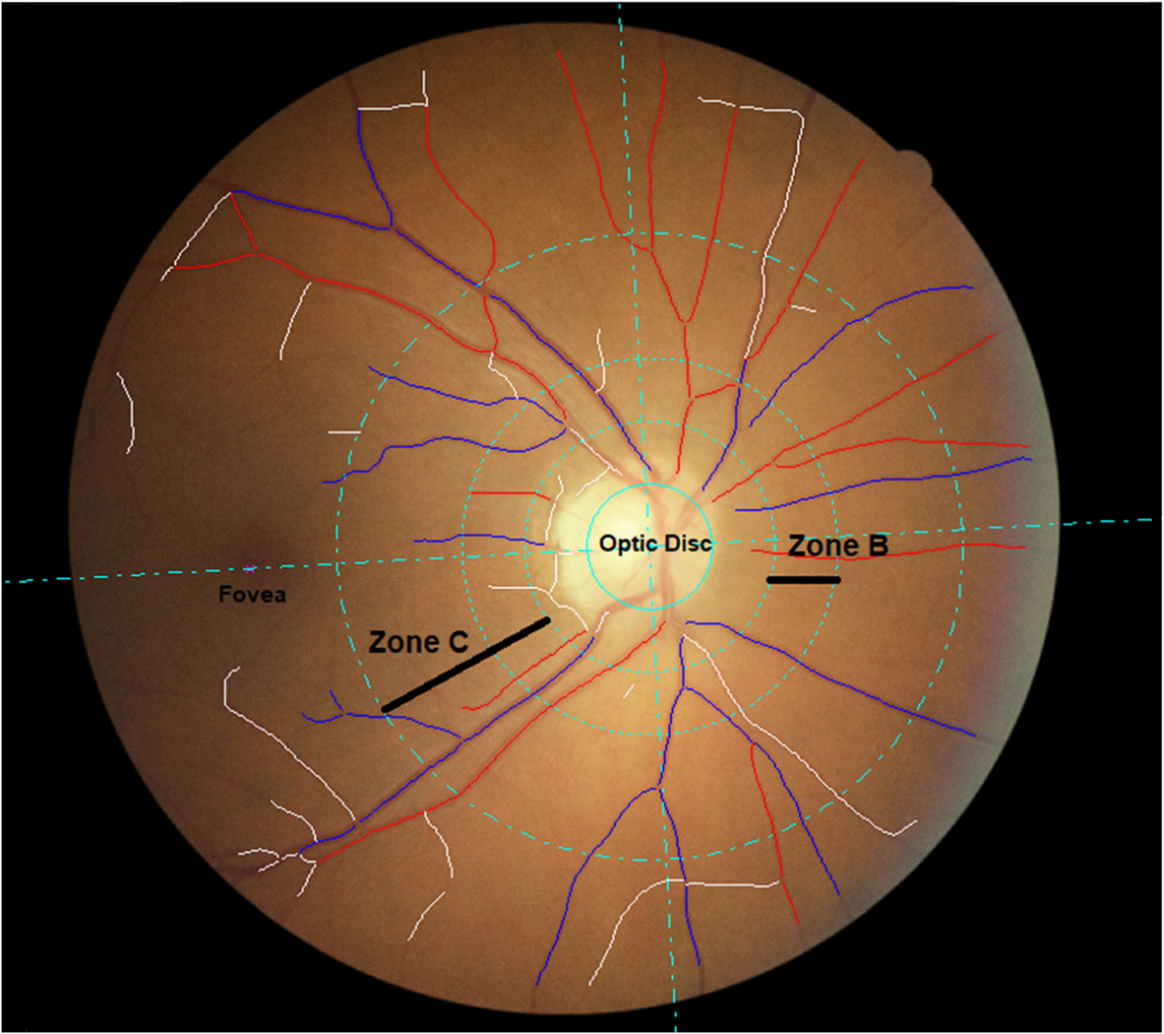
Optic disc centred retinal fundus image assessed using the Vessel Assessment and Measurement Platform for Images of the Retina (VAMPIRE) software. Arterioles (red), venules (blue) and deleted segments (white) are indicated. The retinal microvascular parameters for arteriolar and venular calibre (CRAE, CRVE, and AVR), are calculated from measurements captured in zone B (1.0 to 1.5 optic disc diameters from the centre of the optic disc). Fractal dimension and tortuosity are calculated from measurements captured in zone C (1.0 to 2.5 optic disc diameters from the centre of the optic disc)

Retinal photography was performed through the dilated pupil using a Canon CX-1 Digital Fundus Camera (Canon USA, Melville, NY, USA), following dilatation from a single drop of 1% tropicamide. RMPs (central retinal arteriolar equivalent [CRAE], central retinal venular equivalents [CRVE], arteriolar to venular ratio [AVR], fractal dimension and tortuosity) were measured from optic disc centred fundus images and analysed using the semi-automated software Vessel Assessment and Measurement Platform for Images of the Retina (VAMPIRE; VAMPIRE group, University of Dundee, Dundee, Scotland, Version 3.1, Fig. 1), by a trained grader blinded to participant data [30, 31]. Analysis was undertaken on left eye images except when unavailable or of insufficient quality, in which case the right eye image was used. A paired samples t-test was used to compare a sub-sample of left and right eye measurements from 75 participants. Intraclass correlation coefficients (ICCs) were calculated to assess intergrader reliability with mean values of 0.87 (CRAE) and 0.91 (CRVE).

### Statistical analysis

Analyses were performed using Statistical Package for Social Sciences (Version 24.0. Armonk, NY: IBM Corp). Population characteristics were described using frequencies and percentages for categorical variables or mean and standard deviation (SD) for continuous variables. RMPs were converted into standardised Z-scores, before inclusion in regression models (a standard deviation [SD] increase from the mean). Independent samples t-tests and chi-squared tests were used to compare the distribution of demographic factors and clinical variables between participants according to depression classification. Logistic regression was used to evaluate associations between RMPs and depression status as a binary trait. Minimally adjusted models included age and sex (model 1); with additional adjustment for BMI (model 2) and educational attainment, alcohol consumption, smoking status, CVD, MABP, triglycerides, diabetes, body mass index (BMI) high and low-density lipoprotein (HDL and LDL) levels (model 3). In a sensitivity analysis, participants with diabetes were excluded. *P* < 0.05 was considered statistically significant.

## Results

Data were available for 1376 participants of which 113 (8.2%) and 1263 (91.8%) participants were classified with and without depression (Table 1). Participants had a mean age of 62.0 ± 8.4 yrs. and 52% were female, with 8% categorised as smokers (Table 1). The mean CES-D score for all participants was 5.9 ± 6.4. MABP was 98.2 ± 12.6 mmHg, and 23 and 6% of participants were characterised with diabetes and history of CVD respectively. As expected, individuals with depression had a higher CES-D test score than those without (22.0 ± 6.2 versus 4.4 ± 3.9, respectively). Those classified as free from depression had a higher mean age (62.2 ± 8.4 yrs. versus 59.3 ± 8.1 yrs), a lower percentage were female (52% versus 59%) and they had a lower mean BMI (28.3 ± 4.7 kg/m^2^ versus 29.6 ± 5.4 kg/m^2^) compared to those with depression. Participants with depression were more likely to smoke (12% versus 8%), and a higher proportion had diabetes (29% versus 23%).

**Table 1:** Participant summary characteristics

Participant characteristics	All (***n*** = 1376)	No Depression (***n*** = 1263)	Depression (***n*** = 113)	***P***-Value
Mean age (years, SD)	62.0 ± 8.4	62.2 ± 8.4	59.3 ± 8.1	< 0.01
Female, n (%)	717 (52.1)	650 (51.5)	67 (59.3)	0.11
Smoking status, yes n (%)	115 (8.4)	101 (8.0)	14 (12.4)	0.11
Alcohol consumption, non-drinker, n (%)	280 (20.3)	259 (20.5)	21 (18.6)	0.26
Education, secondary and above, n (%)	1228 (89.2)	1124 (89.0)	104 (92.0)	0.32
Diabetes, yes n (%)	317 (23.0)	284 (22.5)	33 (29.2)	0.10
Mean BMI (kg/m^2^, SD)	28.4 ± 4.8	28.3 ± 4.7	29.6 ± 5.4	< 0.01
Mean arterial blood pressure (mm Hg, SD)	98.2 ± 12.6	98.3 ± 12.6	96.8 ± 12.8	0.24
Cardiovascular disease, no n (%)	1292 (93.9)	1187 (94.0)	105 (92.9)	0.65
Mean triglyceride (mmol/L, SD)	1.6 ± 0.9	1.6 ± 0.9	1.6 ± 0.8	0.50
Mean HDL cholesterol (mmol/L, SD)	1.6 ± 0.5	1.6 ± 0.5	1.6 ± 0.4	0.88
Mean LDL cholesterol (mmol/L, SD)	3.5 ± 1.1	3.4 ± 1.1	3.5 ± 1.1	0.56
Mean CES-D score (SD)	5.9 ± 6.4	4.4 ± 3.9	22.0 ± 6.2	< 0.01

Values are n (%) for categorical variables and mean ± SD for continuous variables. P values were calculated by independent samples t and chi squared tests, P < 0.05 is considered statistically significant. *Abbreviations*: *BMI* body mass index, *HDL* high-density lipoprotein, *LDL* low-density lipoprotein, *SD* standard deviation, *CES-D* The Centre for Epidemiologic Studies Depression Scale

Left and right eye CRAE and CRVE comparisons from 75 participants were not significantly different (P_Crae_ = 0.08; P_Crve_ = 0.89). Only mean arteriolar tortuosity was significantly lower in those with depression in unadjusted comparisons (0.085 versus 0.113; *P* = 0.03; Table 2). Decreased retinal arteriolar tortuosity was associated with depression before and after adjustment for potential confounding variables across all regression models (unadjusted: odds ratio [OR] = 0.81; 95% confidence interval [CI]: 0.67, 0.98; P = 0.03, minimally adjusted: OR = 0.81; 95% CI: 0.67, 0.98; P = 0.03; and fully adjusted: OR = 0.79; 95% CI: 0.65, 0.96; *P* = 0.02); Table 2 and Table 3). No additional significant associations between RMPs and depression were detected (Table 3). In a sensitivity analysis that excluded the 317 participants with diabetes, the effect size of the association observed between arteriolar tortuosity and depression was slightly attenuated and no longer significant given the reduced sample size (Supplementary Table 2).

**Table 2:** Summary of participant retinal microvascular parameters

Retinal microvascular parameters	All (n = 1376)	No Depression (n = 1263)	Depression (n = 113)	P-Value
Mean CRAE (PX, SD)	29.613 ± 2.186	29.636 ± 2.190	29.354 ± 2.136	0.19
Mean CRVE (PX, SD)	40.849 ± 3.314	40.857 ± 3.298	40.757 ± 3.501	0.76
Mean AVR (SD)	0.728 ± 0.062	0.728 ± 0.061	0.724 ± 0.066	0.47
Mean fractal dimension arteriolar (SD)	1.557 ± 0.052	1.557 ± 0.053	1.563 ± 0.046	0.18
Mean fractal dimension venular (SD)	1.539 ± 0.051	1.539 ± 0.051	1.540 ± 0.047	0.89
Mean tortuosity arteriolar (SD)	0.111 ± 0.149	0.113 ± 0.153	0.085 ± 0.090	0.03
Mean tortuosity venular (SD)	0.068 ± 0.109	0.069 ± 0.111	0.063 ± 0.075	0.38

Values are n (%) for categorical variables and mean ± SD for continuous variables. *P* values were calculated by independent samples t tests. *Abbreviations*: *CRAE* central retinal arteriolar equivalent, *CRVE* central retinal venular equivalent, *AVR* retinal arteriolar/venular ratio, *SD* standard deviation, *PX* pixels. *P* < 0.05 was considered statistically significant

**Table 3:** Logistic regression analysis of retinal microvascular parameters and depression

		Model 1			Model 2			Model 3	
Retinal parameter	OR	95%CI	P-Value	OR	95%CI	P-Value	OR	95% CI	P-Value
^a^CRAE (PX)	0.88	0.72, 1.08	0.21	0.89	0.73, 1.09	0.27	0.86	0.70, 1.06	0.17
^a^CRVE (PX)	0.99	0.82, 1.20	0.92	0.98	0.81, 1.19	0.85	0.97	0.79, 1.18	0.75
^a^AVR	0.92	0.75, 1.12	0.40	0.94	0.77, 1.15	0.52	0.92	0.75, 1.13	0.45
^a^Fractal dimension arteriolar	1.12	0.91, 1.37	0.28	1.13	0.92, 1.38	0.24	1.12	0.91, 1.37	0.30
^a^Fractal dimension venular	1.00	0.83, 1.20	0.97	1.00	0.82, 1.20	0.96	0.99	0.82, 1.19	0.89
^ab^Tortuosity arteriolar	0.81	0.67, 0.98	0.03	0.80	0.66, 0.97	0.02	0.79	0.65, 0.96	0.02
^ab^Tortuosity venular	0.93	0.76, 1.13	0.45	0.90	0.74, 1.11	0.33	0.91	0.74, 1.12	0.36

*Abbreviations*: *CRAE* central retinal arteriolar equivalent, *CRVE* central retinal venular equivalent, *AVR* retinal arteriolar/venular ratio, *CI* confidence interval, *OR* odds ratio, *PX* pixels. ^a^RMPs were transformed into standardised Z-scores (based on a SD increase) before inclusion in regression models. ^b^Tortuosity values were skewed and therefore log-transformed before inclusion in regression models. Model 1 was adjusted for age (yrs) and sex; model 2 was adjusted for model 1 covariates plus BMI; model 3 was adjusted for model 2 covariates plus alcohol consumption, smoking status, educational attainment, history of cardiovascular disease, triglycerides, diabetes, mean arterial blood pressure, body mass index, high and low-density lipoprotein levels

## Discussion

We detected significantly lower levels of retinal arteriolar tortuosity, a complexity measure of the twisting and turning of the retinal microvasculature [32], associated with depression independent of potential confounders in older adults. Depression is a condition that affects a significant number of older people, many with underlying chronic illness or cognitive impairment, which leads to disruption of daily life and increased levels of morbidity and mortality. Age and disease related processes, including arteriosclerosis and inflammatory, endocrine, and immune changes, have been associated with the disease processes that characterises depression, especially in those of advanced age [33].

Alexopoulos and colleagues proposed a ‘vascular depression’ hypothesis implicating cerebrovascular disease in the predisposition, precipitation, and perpetuation of geriatric depressive syndromes [28], which provided a rationale for the investigation of associations between RMPs and depression [34]. Previous studies have reported associations between severe and transitory depression and vascular endothelial dysfunction [35–38] but to our knowledge, the current study is the first to report associations between decreased arteriolar tortuosity and later-life depression. Endothelial cells also play a key role in regulating retinal microvascular blood flow and angiogenesis and our findings may implicate microvascular endothelial dysfunction and reduced retinal arteriolar tortuosity and in older people with depression independent of potential confounding factors. Indeed, a recent systematic-review and meta-analyses reported associations between peripheral and cerebral forms of microvascular dysfunction with an increased odds of incident late-life depression supporting the hypothesis that microvascular dysfunction is causally linked to depression and a potential target for the prevention and treatment of symptoms [39]. The meta-analysis by van Agtmaal and colleagues evaluated several outcome measures including retinal vessel calibre [23, 40], detecting no association with depression, in line with the findings from our study.

Technological advances in non-invasive retinal image acquisition and analysis have enabled more accurate quantification of microvascular health [17]. A small number of studies investigating associations between RMPs and depression have reported inconsistent findings [23–27]. Furthermore, these studies were largely limited to the assessment of retinal vessel calibre only and were unable to consider other RMPs such as fractal dimension or tortuosity, coefficients that reflect the status of microvascular health. Sun and colleagues reported no significant associations between retinal microvascular calibre and depression similarly characterised using the CES-D questionnaire in 2420 individuals ≥65 yrs. old in the population-based Cardiovascular Health Study [26]. The Rotterdam population-based study also characterised depression using either the CES-D questionnaire or the Hospital Anxiety and Depression Scale, and reported no significant associations between retinal microvascular calibre and late-life depression in 3605 participants ≥55 yrs. [23]. Kim and colleagues identified retinal arteriolar narrowing in individuals characterised with depression using the CES-D questionnaire in a cross-sectional study of 1744 older adults (mean age of 78 yrs) from the Cardiovascular Health Study [27]. Other forms of depression and age categories have also been considered. Li and colleagues measured symptoms of antenatal anxiety and depression using the State-Trait Anxiety Inventory and Edinburgh Postnatal Depression Scale reporting associations between wider retinal arterioles and depression in 952 pregnant women [25]. Wider retinal arterioles were also reported by Meier and colleagues in 865 adolescents and young adults from the Brisbane Longitudinal Twin Study and the Twin Eye Study with symptoms of anxiety characterised by the Somatic and Psychological Health Report questionnaire, with the authors suggesting this may implicate microvascular variation as an early mechanistic factor in depression disease aetiology [24], in contrast to arteriolar narrowing described by Kim and colleagues in older adults [24, 27].

Our study had several limitations. Firstly, the study population consisted of Caucasian participants, aged > 50 yrs. and may represent the ‘worried-well’, limiting the generalisability of our results. Secondly, although NICOLA is a longitudinal study, only baseline data were available for this cross-sectional analysis precluding assumptions regarding causality. Thirdly, although adjustment for a large number of potential confounders was made, the possibility of residual confounding remains. Additionally, despite adjusting for cardiometabolic, atherosclerotic and diabetic risk factors, we cannot fully discount their potential influence on our findings, given previously reported associations [41–45].

Despite the limitations, our study had several strengths. Similar to previous investigations of RMPs and depression [23–27], optic disc centred fundus images provided a more accurate quantification of RMPs compared to macula centred images, which generally limit assessment to the temporal retinal arcades. Although our analysis was cross-sectional in design, it provides a rationale for further evaluation of RMPs in future waves of NICOLA data. If clinical value is associated with identifying those individuals at increased risk of depression by using RMPs, earlier identification of older individuals at increased risk of adverse vascular events associated with depression may be possible. In this study, retinal images from the left eye were analysed except when unavailable or of insufficient quality, in which case the right eye image was used. This was unlikely to have limited the study conclusions, as comparable investigations have previously reported high correlations between RMPs from right and left eye comparisons [46–48], similar to the comparisons undertaken in a subset of NICOLA participants.

Furthermore, the well-characterised population-based study design added validity to our findings. Finally, the CES-D questionaire to classify depression is well accepted with previously reported sensitivity and specificity values of 0.87 and 0.70, respectivly [49] and the exclusion of participants using anti-depressive medications or with MCI improved the characterisation of depression, given those with depressive symptoms are more likely to score poorly on tests of cognitive function [50].

In conclusion, we report decreased retinal arteriolar tortuosity in association with depression in an older population independent of potential confounding factors. These retinal measures may provide non-invasive assessment of microvascular complications associated with depression.

## Supplementary Information

**Additional file 1.**

## Data Availability

The data that support the findings of this study are available from the Northern Ireland Cohort for the Longitudinal Study of Ageing but restrictions apply to the availability of this data, which was used under license for the current study, and so is not publicly available. Data may however be available from the corresponding authors upon reasonable request and provided there is permission from NICOLA.

## Abbreviations

NICOLA: The Northern Ireland Cohort for the Longitudinal study of Ageing
BMI: Body mass index
HDL: High-density lipoprotein
LDL: Low-density lipoprotein
CRAE: Central retinal arteriolar equivalent
CRVE: Central retinal venular equivalent
AVR: Retinal arteriolar/venular ratio
SD: Standard deviation
PX: Pixels
SBP: Systolic blood pressure
DBP: Diastolic blood pressure
CI: Confidence intervals
β: beta value
VAMPIRE: Vessel Assessment and Measurement Platform for Images of the Retina
MABP: Mean arteriole blood pressure
RMPs: Retinal microvascular parameters
Yrs: Years
CVD: Cardiovascular disease
CAPI: Computer-aided personal interview
NI: Northern Ireland
CES-D: The Centre for Epidemiologic Studies Depression Scale
MCI: Mild cognitive impairment
