# Supplementary material for: Association of reduced retinal arteriolar tortuosity with depression in older participants from the Northern Ireland Cohort for the Longitudinal Study of Ageing
Source: BMC Geriatr. 2021 Jan 15;21:62. doi: 10.1186/s12877-021-02009-z (PMC7809811; doi:10.1186/s12877-021-02009-z)
Supplement: Supplementary file 1 — Additional file 1. [file 12877_2021_2009_MOESM1_ESM.docx]

**Title Page:**

Association of reduced retinal arteriolar tortuosity with depression in older participants from the Northern Ireland Cohort for the Longitudinal Study of Ageing

**Authorship**

O’Neill RA^1^, Maxwell AP^1^, Kee F^1^, Young I^1^, Hogg RE^1^, Cruise S^1^, McGuinness B^1^, McKay GJ^1†^

**Author Affiliations**

^1^Centre for Public Health, Queens University Belfast, Belfast, Northern Ireland

^†^Corresponding author:

Gareth McKay

Centre for Public Health

Queens University Belfast

g.j.mckay@qub.ac.uk

**Supplementary Table 1**: Comparison of demographic characteristics between all participants with retinal fundus imaging with and without VAMPIRE retinal measures.

| **Participant characteristics** | **Participants with images included (n=1,376)** | **Participants with images excluded (n=709)** | **P- value** |
| --- | --- | --- | --- |
| Mean age (years, SD) | 62.0 ± 8.4 | 66.0 ± 8.9 | <0.01 |
| Female, n (%) | 717 (52.1) | 325 (45.8) | <0.01 |
| Smoking status, yes n (%) | 115 (8.4) | 72 (10.2) | 0.17 |
| Alcohol consumption, non-drinker, n (%) | 280 (20.3) | 173 (24.4) | 0.08 |
| Education, secondary and above, n (%) | 1228 (89.2) | 609 (85.9) | 0.03 |
| Diabetes, yes n (%) | 317 (23.0) | 107 (15.1) | <0.01 |
| Mean BMI (kg/m^2^, SD) | 28.4 ± 4.8 | 28.8 ± 4.7 | 0.07 |
| Mean arterial blood pressure (mm Hg, SD) | 98.2 ± 12.6 | 100.0 ± 12.7 | <0.01 |
| Cardiovascular disease, no n (%) | 1292 (93.9) | 638 (90.0) | <0.01 |
| Mean CES-D score (SD) | 5.9 ± 6.4 | 6.5 ± 6.8 | 0.03 |
| Depression, yes n (%) | 113 (8.2) | 65 (9.2) | 0.46 |
| Depression, no n (%) | 1263 (91.8) | 644 (90.8) | 0.46 |

Values are n (%) for categorical variables and mean ± SD for continuous variables. P < 0.05 is considered statistically significant. P values were calculated by independent samples t and chi squared tests, P < 0.05 is considered statistically significant. Abbreviations: BMI, body mass index; HDL, high-density lipoprotein; LDL, low-density lipoprotein; SD, standard deviation; CES-D, The Centre for Epidemiologic Studies Depression Scale.

**Supplementary Table 2**. Sensitivity analysis of binary logistic regression analysis of retinal microvascular parameters and depression following the exclusion of the 317 participants with diabetes.

|  |  | **Model 1** |  |  |  | **Model 2** |  |  |  | **Model 3** |  |
| --- | --- | --- | --- | --- | --- | --- | --- | --- | --- | --- | --- |
| **Retinal parameter** | **OR** | **95%CI** | **P-Value** |  | **OR** | **95%CI** | **P-Value** |  | **OR** | **95% CI** | **P-Value** |
| ^a^CRAE (PX) | 0.98 | 0.77, 1.24 | 0.87 |  | 1.01 | 0.80, 1.29 | 0.93 |  | 0.99 | 0.77, 1.27 | 0.92 |
| ^a^CRVE (PX) | 1.01 | 0.80, 1.28 | 0.93 |  | 1.01 | 0.80, 1.28 | 0.94 |  | 1.00 | 0.79, 1.27 | 0.99 |
| ^a^AVR | 0.98 | 0.77, 1.23 | 0.84 |  | 1.00 | 0.79, 1.27 | 0.98 |  | 0.99 | 0.78, 1.26 | 0.95 |
| ^a^Fractal dimension arteriolar | 1.14 | 0.89, 1.45 | 0.30 |  | 1.15 | 0.91, 1.47 | 0.25 |  | 1.15 | 0.90, 1.46 | 0.28 |
| ^a^Fractal dimension venular | 0.99 | 0.79, 1.23 | 0.90 |  | 0.99 | 0.79, 1.24 | 0.92 |  | 0.98 | 0.78, 1.24 | 0.90 |
| ^ab^Tortuosity arteriolar | 0.83 | 0.66, 1.05 | 0.12 |  | 0.82 | 0.65, 1.04 | 0.10 |  | 0.82 | 0.65, 1.04 | 0.10 |
| ^ab^Tortuosity venular | 0.87 | 0.69, 1.11 | 0.27 |  | 0.85 | 0.67, 1.09 | 0.20 |  | 0.86 | 0.67, 1.10 | 0.23 |

Abbreviations: CRAE, central retinal arteriolar equivalent; CRVE, central retinal venular equivalent; AVR, retinal arteriolar/venular ratio; CI, confidence interval; OR, odds ratio; PX, pixels. ^a^RMPs were transformed into standardised Z-scores (based on a SD increase) before inclusion in regression models. ^b^Tortuosity values were skewed and therefore log-transformed before inclusion in regression models. Model 1 was adjusted for age (yrs) and sex; model 2 was adjusted for model 1 covariates plus BMI; model 3 was adjusted for model 2 covariates plus alcohol consumption, smoking status, educational attainment, history of cardiovascular disease, triglycerides, diabetes, mean arterial blood pressure, body mass index, high and low-density lipoprotein levels.

**Supplementary Figure 1:** A flow chart of participant inclusion and exclusion criteria.

Included

Excluded

Supplementary analysis: Participants without retinal measure and with CES-D data and absence of MCI or use of anti-depressive medications

(n=709)

Supplementary analysis: Participants without retinal and CES-D data and absence of MCI or use of anti-depressive medications

(n= 680)

Excluded

2^nd^ Step: Participants without retinal measures

(n= 1,389)

3^rd^ Step: Participants with retinal measures and CES-D data and absence of MCI or use of anti-depressive medications

(n= 1,376)

3^rd^ Step: Participants without measures of depression or had MCI or use of anti-depressive medications

(n= 753)

NICOLA participants

All (n= 8,468)

Final participants included within the study

(n= 1,376)

Included

Excluded

Included

2^nd^ Step: Participants with retinal measures

(n= 2,129)

Excluded

Included

1^st^ Step: Participants without retinal images

(n= 4,950)

1^st^ Step: Participants with retinal images

(n= 3,518)
